# Self-Organized Synchronous Calcium Transients in a Cultured Human Neural Network Derived from Cerebral Organoids

**DOI:** 10.1016/j.stemcr.2019.05.029

**Published:** 2019-06-27

**Authors:** Hideya Sakaguchi, Yuki Ozaki, Tomoka Ashida, Takayoshi Matsubara, Naotaka Oishi, Shunsuke Kihara, Jun Takahashi

**Affiliations:** 1 Department of Clinical Application, Center for iPS Cell Research and Application, Kyoto University, Kyoto 606-8507, Japan; 2 Life Science Center, MB HQ, Yokogawa Electric Corporation, Ishikawa 920-0177, Japan; 3 Informatics Japan, PerkinElmer Japan Co., Ltd., Tokyo 101-0024, Japan; 4 Department of Fundamental Cell Technology, Center for iPS Cell Research and Application, Kyoto University, Kyoto 606-8507, Japan

**Keywords:** cerebral organoid, human pluripotent stem cell, three-dimensional imaging, calcium imaging, neural network activity

## Abstract

The cerebrum is a major center for brain function, and its activity is derived from the assembly of activated cells in neural networks. It is currently difficult to study complex human cerebral neuronal network activity. Here, using cerebral organoids, we report self-organized and complex human neural network activities that include synchronized and non-synchronized patterns. Self-organized neuronal network formation was observed following a dissociation culture of human embryonic stem cell-derived cerebral organoids. The spontaneous individual and synchronized activity of the network was measured via calcium imaging, and subsequent analysis enabled the examination of detailed cell activity patterns, providing simultaneous raster plots, cluster analyses, and cell distribution data. Finally, we demonstrated the feasibility of our system to assess drug-inducible dynamic changes of the network activity. The comprehensive functional analysis of human neuronal networks using this system may offer a powerful tool to access human brain function.

## Highlights

• Cerebral organoids can recapitulate cerebral characters in 3D order
• A functional neural network was efficiently formed after dissociation of organoids
• Calcium activity patterns were examined with clustering and the cell distribution
• A powerful system for the functional analysis of human neuronal networks

## Graphical Abstract

**Figure:**
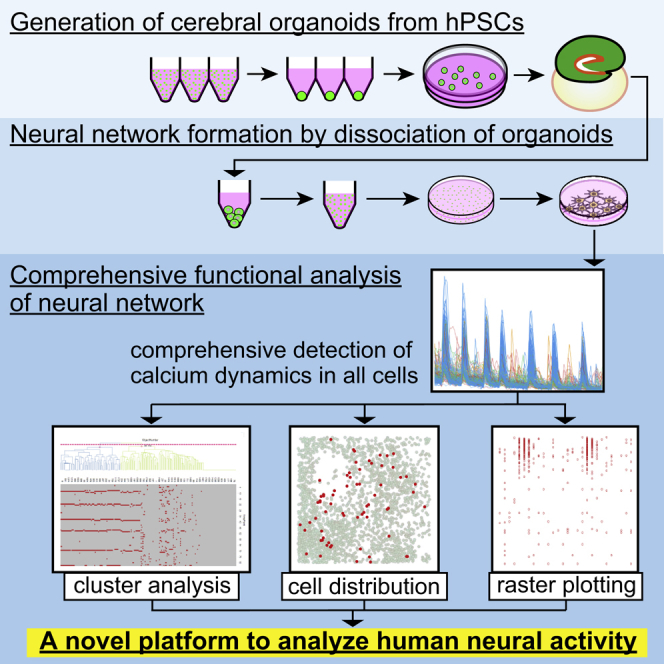

## Teaser

Cerebral activity is derived from the assembly of activated cells, but it is currently difficult to study human cerebral neuronal network activities. Here, Sakaguchi et al. report self-organized and complex human neural network activity using organoid technology and drug-inducible dynamic changes of the activity that will be useful for future research on human brain function and neuropsychiatric disorders.

## Introduction

The cerebrum is the largest and most complex tissue with complexed neural activity (Northcutt and Kaas, 1995). The cerebrum localizes to the most rostral side of the neural system and is particularly developed in primates (Lui et al., 2011). The function of the cerebrum is wide ranging, especially in humans, and covers motor, sensory, visual, auditory, and higher-ordered brain functions such as intention and memory (Lui et al., 2011). These neural functions are thought to depend on distinct patterns of the network activity in cell assemblies (Luczak et al., 2015, Spatz, 1996).

Recent progress in stem cell technology has enabled the induction of cerebral tissues from human pluripotent stem cells (hPSCs) in three dimensions (3D) (Kadoshima et al., 2013, Lancaster et al., 2013, Sasai, 2013a, Sasai, 2013b). Notably, cerebral organoids, which are stem cell-derived cerebral structures generated *in vitro* that display features of the 3D architecture and physiology of the cerebrum organ, have paved a novel way to approach and analyze human cerebral tissues (Bershteyn et al., 2017, Birey et al., 2017, Dang et al., 2016, Garcez et al., 2016, Kadoshima et al., 2013, Lancaster et al., 2013, Lancaster et al., 2017, Qian et al., 2016, Quadrato et al., 2017, Watanabe et al., 2017, Xiang et al., 2017). Since cerebral organoids have the potential to recapitulate at least partially the developmental process of cerebrum formation in 3D, they have enabled the modeling of not only human cerebral development but also cerebrum-related diseases such as microcephaly, Zika virus infection, glioblastoma, and Timothy syndrome (Birey et al., 2017, Dang et al., 2016, Garcez et al., 2016, Kadoshima et al., 2013, Lancaster et al., 2013, Ogawa et al., 2018, Qian et al., 2016, Quadrato et al., 2017, Watanabe et al., 2017). Despite these recent technical breakthroughs, current cerebral organoid technologies still have significant limitations, especially regarding the functional evaluation of neural network activity, which is indispensable for the examination of human brain function or the modeling of neuropsychiatric disorders. Although some recent reports have utilized calcium imaging for the characterization of cerebral organoids (Bershteyn et al., 2017, Lancaster et al., 2017, Mansour et al., 2018, Watanabe et al., 2017, Xiang et al., 2017) including the use of high-density silicon microelectrodes to prove network activity in organoids (Mansour et al., 2018, Quadrato et al., 2017), detailed evaluation of the activity in human neural networks has not been achieved.

In the present study, we evaluated individual and synchronized patterns of human cerebral neural network activity. To this end, we efficiently generated cerebral organoids, characterized them by 3D imaging, and dissociated them to create self-organized neuronal networks that were evaluated via time-lapse imaging. Imaging intracellular calcium dynamics revealed that cells in human neural networks showed synchronized bursts with some spontaneous individual activities *in vitro*. In addition, we assessed the neural network functionality via drug-induced dynamic modulation of the network activity. Overall, our findings suggest that our approach may allow investigation of the functionality of human neuronal networks.

## Results

### Induction of Cerebral Organoids with Elongated Epithelium

**Figure 1:**
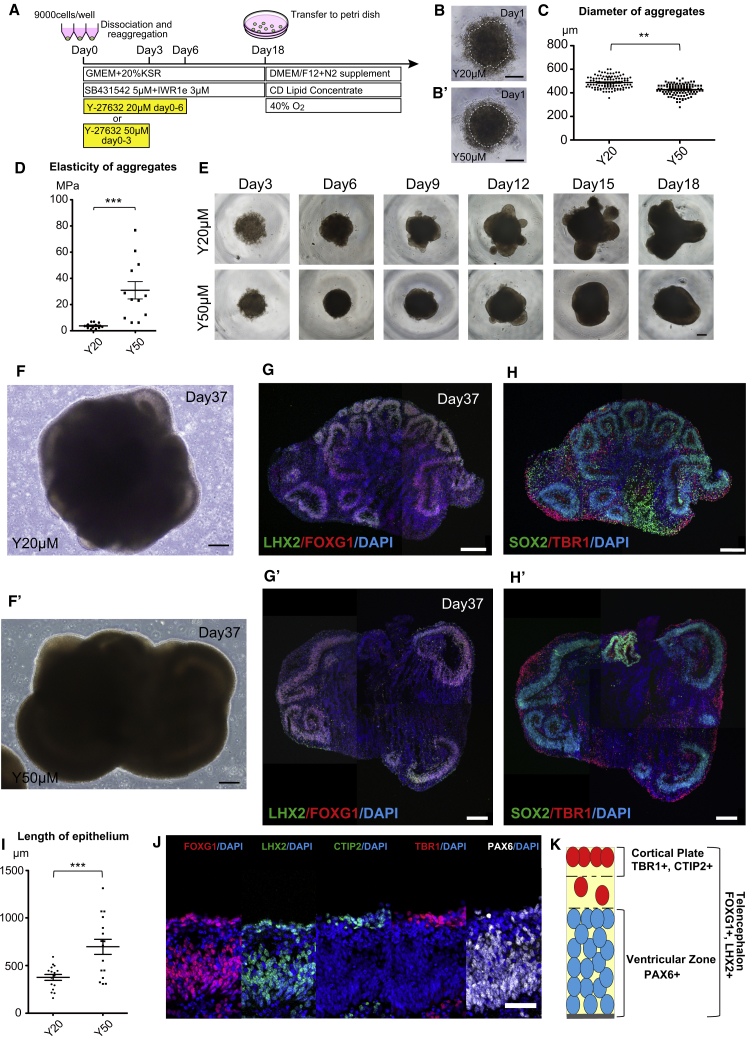
Generation of Cerebral Organoids with Elongated Epithelium from hESCs (A) Schematic of conditions used to induce cerebral organoids. (B and B′) Phase-contrast image of cell aggregation induced from hESCs on day 1 under the Y20 condition (Y-27632 20μM) (B) and Y50 condition (Y-27632 50μM) (B′). White dashed circles indicate the morphology of the aggregates. (C) Comparison of diameters of aggregates on day 1. The diameter was significantly smaller under the Y50 condition. Sample number: 106 aggregates from 8 independent experiments for Y20, and 110 aggregates from 11 independent experiments for Y50 conditions. ^∗∗^p < 0.01, unpaired t test. (D) Elasticity of the aggregates under each condition examined by AFM. n = 9 (number of counted spots was 3/aggregate and 3 samples per condition). ^∗∗∗^p < 0.001, unpaired t test. (E) Phase-contrast image of Y20 and Y50 conditions on days 3, 6, 9, 12, 15, and 18. (F and F′) Phase-contrast images of Y20 and Y50 conditions on day 37. (G–H′) IHC for LHX2 and FOXG1 (G and G′) and SOX2 and TBR1 (H and H′) in day-37 aggregates under each condition. A more elongated epithelium was formed under the Y50 condition. (I) Comparison of overall epithelium length between each condition. n = 16 from 4 aggregates for each condition. One cricoid-like epithelium is counted as n = 1. ^∗∗∗^p < 0.001, unpaired t test. (J) IHC for cortical markers FOXG1, LHX2, CTIP2, TBR1, and PAX6 under the Y50 condition. (K) Schematic image of cerebral epithelium induced under the Y50 condition. Error bars in graphs denote SEM. Nuclear counterstaining (blue), DAPI. See also Figures S1 and S2; Video S1. Scale bars, 200 μm (B, E, and F–H′) and 50 μm (J).

For the induction of 3D neural tissues, we adapted SFEBq (serum-free floating culture of embryoid body-like aggregates with quick reaggregation), the pioneering method by Yoshiki Sasai and colleagues (Kadoshima et al., 2013, Watanabe et al., 2005, Eiraku et al., 2008, Eiraku et al., 2011, Sakaguchi et al., 2015). Despite being able to recapitulate cerebral tissue induction via the protocol originally described by Kadoshima et al. (2013), we found the size of the neuroepithelium in the induced aggregates to be not as extensive as previously reported. Since incomplete morphological differentiation is one of the major problems in organoid research (Lancaster et al., 2017), we first sought to overcome this critical hurdle. We noticed that the surface of the aggregates was rough, resulting in incomplete aggregation at the early stages of differentiation under the previous method (Figures 1A, 1B, and S1A; 20 μM Y-27632). We thus hypothesized that more homogeneous and dense cell aggregations may generate neural epithelium more robustly. Because Y-27632, a specific inhibitor of Rho-dependent protein kinase (ROCK), is known to prevent actomyosin hyperactivation and apoptosis in dissociated hPSCs (Ohgushi et al., 2010, Watanabe et al., 2007), we further modified the original differentiation method by adding a higher concentration of Y-27632 (50 μM from day 0 to day 3, cited as the Y50 condition) and compared it with the original (Y-27632, 20 μM from day 0 to day 6; cited as the Y20 condition) (Figure 1A).

Under the Y50 condition, the aggregates became round and had a smooth surface, which was not seen in the Y20 condition (Figures 1B, 1B′, S1A, and S1A′). Bright-field view imaging showed this phenomenon occurred from 6 h after the start of differentiation (Video S1). Although the total cell number at the start of the induction was the same, the diameters of the aggregates at day 1 (Figure 1C) and day 3 (Figure S1B) were significantly smaller in the Y50 condition than the Y20 condition, suggesting denser cell aggregation under the Y50 condition. To test the physiological properties of the aggregates, we adopted atomic force microscopy (AFM) analysis (Eiraku et al., 2011, Mackay and Kumar, 2013) (Figures S2A–S2C). AFM enabled us to measure the elasticity of the induced aggregates, revealing significantly higher elasticity in aggregates under the Y50 condition, which is consistent with a more dense and tight cell aggregation (Figures 1D and S2D–S2D″). As the culture period progressed, the shape of the aggregates remained round under the Y50 condition, while a variety of shapes were observed in the Y20 condition (Figure 1E). At day 37, elongated neuroepithelia were clearly observed in the Y50 condition, while the epithelium in the Y20 condition remained short (Figures 1F, 1F′, and S1C–S1D′). Immunohistochemistry (IHC) analysis at day 37 showed telencephalic marker FOXG1 and dorsal telencephalic marker LHX2 expression in the neuroepithelium under both conditions (Figures 1G and 1G′). The expression patterns of the mitotic progenitor marker SOX2 and of the postmitotic cortical layers 1 and 6 marker TBR1 were clearly separated (Figures 1H and 1H′). The expression pattern of N-CADHERIN (NCAD), which is known to be expressed higher in the apical side of the epithelium, was consistent with the formation of an elongated neuroepithelium under the Y50 condition (Figures S1E and S1E′). Although marker expression patterns were the same under the Y20 condition, the length of the epithelium was significantly smaller (Figures 1G–1I), suggesting that the Y50 condition is more suitable for the generation of an elongated cortical epithelium. More detailed IHC analysis regarding cortical layer formation in the Y50 condition showed that telencephalic markers FOXG1 and LHX2 were expressed in the entirety of the epithelium, while the dorsal telencephalic progenitor marker PAX6 was expressed on the apical side of the ventricular zone (VZ), and that TBR1 and cortical layers 5 and 6 marker CTIP2 were expressed in the cell layer outside of the VZ (Figure 1J). These characteristic expression patterns are consistent with cortical plate (CP) and VZ formations described previously (Kadoshima et al., 2013) (Figure 1K).

Video S1. Bright-Field View Imaging of Cell-Aggregation Process from Days 0 to 3, Related to Figure 1 Video corresponds to Figures 1B, 1B′, S1A, and S1A′, and explanatory text in the Results section.

We also applied this method to human induced pluripotent stem cells (hiPSCs) (Figure S1F). At day 37, the neuroepithelium was clearly generated in hiPSC-derived aggregates (Figure S1G). IHC analysis also showed FOXG1^+^/LHX2^+^/NCAD^+^ telencephalic tissue generation (Figures S1H–S1J), SOX2^+^/PAX6^+^ VZ formation (Figures S1K and S1L), and CTIP2^+^/TBR1^+^ CP formation (Figures S1M and S1N). Collectively, our modified method successfully generated cerebral organoids from hPSCs.

### 3D and Functional Analysis of Long-Term Cultured Cerebral Organoids

**Figure 2:**
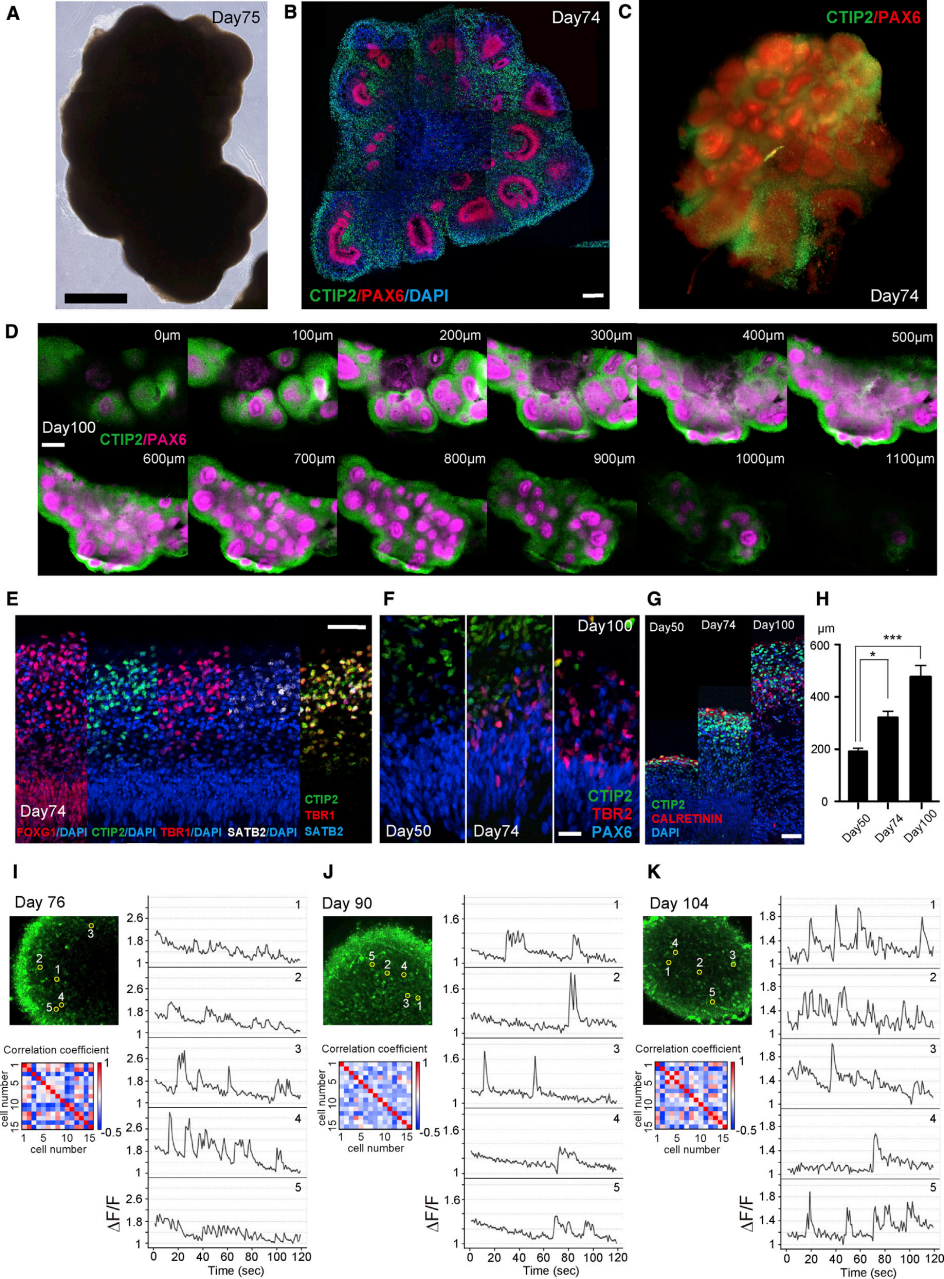
3D Imaging and Functional Analysis of Cerebral Organoids (A) Phase-contrast image of a cerebral organoid on day 75 under the Y50 condition. (B) IHC for CTIP2 and PAX6 on day 74. (C) 3D reconstruction view of a whole aggregate using light sheet microscopy. (D) 3D z-stack imaging of CTIP2/PAX6 IHC for a whole aggregate on day 100. The thickness of the detected zone exceeded 1,000 μm. (E) IHC for FOXG1, CTIP2, TBR1, and SATB2 of day-74 aggregates. (F) IHC for CTIP2, TBR2, and PAX6 of day-50, -74, and -100 aggregates. (G) IHC for CTIP2 and CALRETININ of days 50, 74, and 100. (H) Thickness of total epithelium on days 50, 74, and 100. The total thickness was significantly thicker on days 74 and 100 compared with day 50. n = 10 from 6 aggregates for day 50, and n = 10 from 5 aggregates for days 74 and 100. One cricoid-like epithelium is counted as n = 1. ^∗^p < 0.05, ^∗∗∗^p < 0.001, one-way ANOVA. (I–K) Calcium imaging datasets on day 76 (I), 90 (J), and 104 (K). Left upper image: representative image of active neurons. Right panel: the firing pattern of selected cells shown by trace image of the calcium response. Left lower image: heatmap of the correlation coefficients between 15 cells in an image. The data are representative of three independent experiments. Error bars in graphs denote SEM. Nuclear counterstaining (blue), DAPI. Scale bars, 1,000 μm (A), 200 μm (B and D), 50 μm (E and G), and 20 μm (F). See also Figure S3 and Videos S2, S3, and S4.

Our modified method enabled the long-term culture of cerebral organoids under simple culture conditions (Figure S3A). After culturing in medium with N2 supplement and cutting them into halves or thirds at around day 35, cerebral organoids grew in size while retaining a clear epithelium (Figures 2A, S3A, and S3B). IHC of PAX6 and CTIP2 showed clear formation of the cerebral epithelium at day 74 (Figure 2B). To further characterize the generated cerebral organoids, we conducted a 3D evaluation of whole aggregates. Following tissue rarefaction via the CLARITY system with some modifications of the original method (Kim et al., 2015), we performed 3D IHC and imaging of whole organoids using light sheet microscopy, which revealed the generation of multiple PAX6^+^ cricoid-like progenitor zones and surrounding CTIP2^+^ CP layers in a single aggregate at day 74 (Figure 2C and Video S2). z-Stack imaging of day-100 samples by confocal microscopy also confirmed the generation of multiple PAX6^+^ cricoid-like structures and CTIP2^+^ post mitotic neural zones surrounding them (Figure 2D and Video S3). These findings confirmed the effective generation of cerebral organoids in a whole aggregate.

Video S2. 3D Imaging of a Whole Organoid at Day 74 Using Light Sheet Microscopy, Related to Figure 2 Video corresponds to Figure 2C, and explanatory text in the Results section.

Video S3. Z-Stack Imaging of a Whole Organoid at Day 100 Using Confocal Microscopy, Related to Figure 2 Video corresponds to Figure 2D, and explanatory text in the Results section.

Next, we analyzed in detail the character of the induced long-term cultured neuroepithelial structures. At day 74, in the FOXG1^+^ telencephalic epithelium, a CP-like layer positive for CTIP2, TBR1, and an upper layer marker, SATB2, was formed (Figure 2E). The subventricular zone (SVZ) layer positive for the intermediate progenitor marker TBR2 was prominent in the outer side of PAX6^+^ VZ after day 74 (Figure 2F). The thickness of the neuroepithelium increased along with the culture period (Figures 2G, 2H, and S3C). The thickness of both the CTIP2^+^/TBR1^+^ deep layer zone and SATB2^+^ upper layer zone also significantly increased along with the progression of the culture period (Figure S3D). SOX2^+^/phosphorylated-VIMENTIN (pVIM)^+^ outer-radial glia-like cells were seen from culture day 50 to day 100, and these cells tended to localize farther away from the apical side as the culture period proceeded (Figure S3E). These data indicate typical characteristic marker expression of dorsal telencephalon including VZ, SVZ, deep cortical layer, and upper cortical layer with recapitulation of not only several developmental aspects of cortical layer formation but also the character of radial glia cells.

We next examined the neural functionality of these 3D cerebral tissues. For this purpose, we detected intracellular calcium dynamics of the organoids using two-photon microscopy. At day 76, spontaneous calcium dynamics could be detected in the aggregates, and almost all cells showed asynchronous calcium transients (Figure 2I and Video S4). The low value of the correlation coefficient between 15 cells in three fields indicated little synchronized activity (mean correlation coefficient value: 0.160 ± 0.100). We subsequently detected the calcium activity of day-90 and day-104 samples, which revealed no significant change in the activity pattern regarding synchronicity (mean correlation coefficient: 0.063 ± 0.062 [day 90] and 0.171 ± 0.092 [day 104]) (Figures 2J and 2K; Video S4). This functional analysis showed spontaneous individual activity in cerebral organoids from days 76–104 with little synchronized activity, suggesting that the neural activity in 3D organoids may be immature.

Video S4. Calcium Imaging of a Cerebral Organoid Using Multi-Photon Microscopy at Days 76, 90, and 104, Related to Figure 2 Video corresponds to Figures 2I–2K, and explanatory text in the Results section.

### Self-Organized Neural Network Formation by Dissociation of Cerebral Organoids

**Figure 3:**
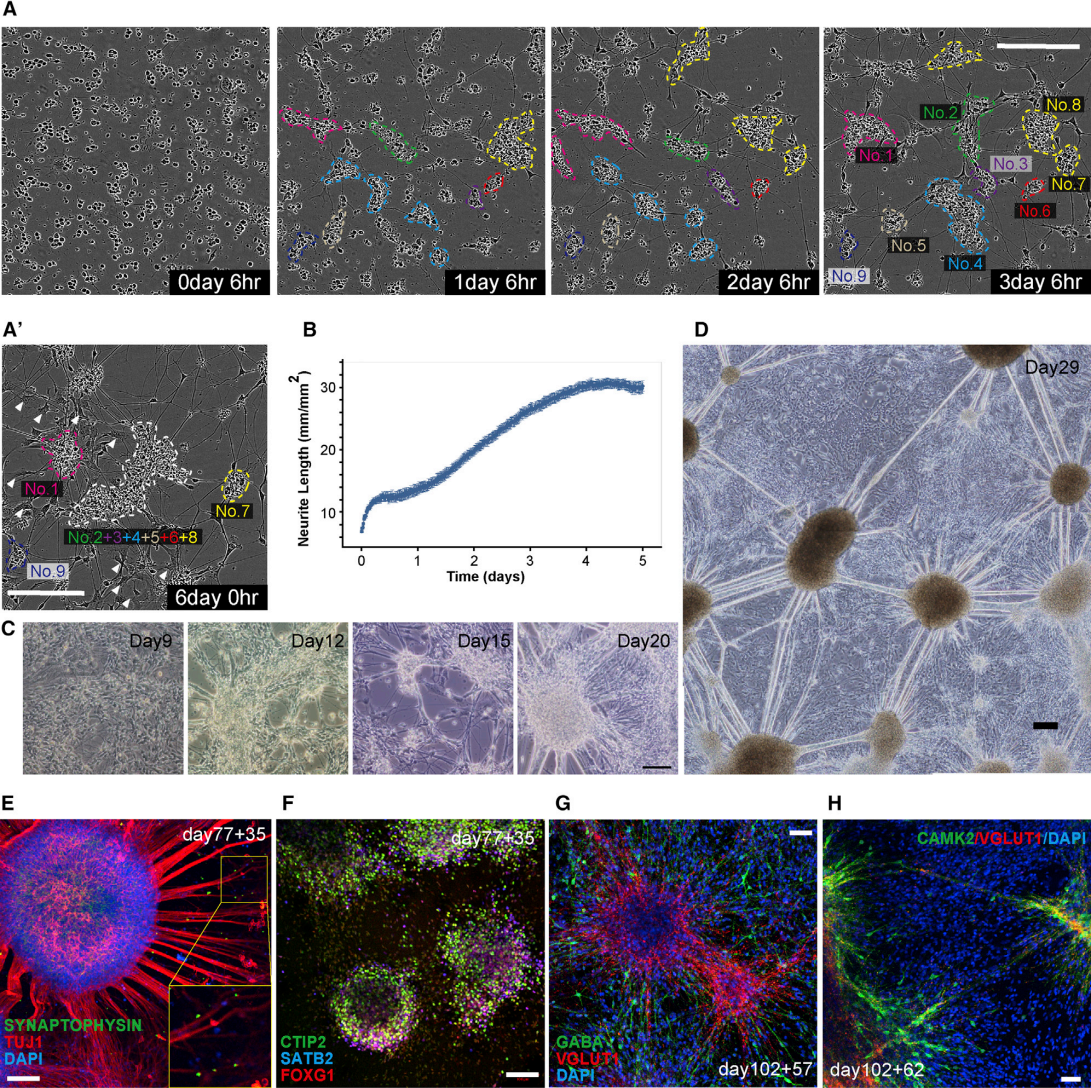
Self-Organized Neural Network Formation by Dissociation of Cerebral Organoids (A) Time course of phase-contrast images after dissociation of cerebral organoids. Several small cell clusters were formed. These clusters formed axonal connections during their movement via fuse-and-separate motion (1 day 6 h to 3 days 6 h). (A′) At 6 days after dissociation, some clusters fused and made a big cell cluster that formed a broad axonal network (indicated by the cluster numbers described in A). Some glial-like shaped cells were generated from 100 h after dissociation (white arrowheads in A′). (B) Neurite extension analysis at 15-min intervals for 5 days. The data are the average values of 16 fields in one well of a 24-well plate taken by a 10× objective lens. (C) Phase-contrast images of dissociated organoids 9, 12, 15, and 20 days after dissociation. The neural connections gradually became thicker. (D) Phase-contrast image of a neuronal network 29 days after dissociation. A vast and thick neural network was formed on glial-like cells. (E) IHC for TUJ1 and SYNAPTOPHYSIN 35 days after dissociation. (F) IHC for CTIP2, SATB2, and FOXG1 35 days after dissociation. (G) IHC for VGlut1 and GABA 57 days after dissociation. (H) IHC for VGlut1 and CAMK2 62 days after dissociation. Error bars in graphs denote SEM. Nuclear counterstaining (blue), DAPI. Scale bars, 200 μm (A, A′, and D), 100 μm (C, E, and F), and 50 μm (G and H). See also Figure S4 and Video S5.

To achieve a longer culture of the neural network, we adopted a dissociation culture of *in vitro* formed 3D tissues based on rodent studies (Chiappalone et al., 2006). Cerebral organoids were dissociated at days 70–100 and plated on poly-D-lysine-laminin-fibronectin-coated plates (Figure 3A, 0 day 6 h, Figure S4A). After dissociation into single cells, several small cell clusters were formed, some of which showed active migration (Figure 3A, 1 day 6 h). Besides random axonal elongation from 1 day after dissociation, axonal connections were established when several clusters fused and separated (Figure 3A, 2 days 6 h to 3 days 6 h). Following repeated fuse-and-separation motion cycles, a network between each cluster was tightly formed, and starting from 100 h after dissociation, glial-like shaped cells were continuously generated (Figure 3A′ and Video S5). Analysis of the neurites showed increased neurite extensions in the first 4–5 days after dissociation (Figures 3B and S4B). Neural connections gradually became thicker with progression of the culture period (Figure 3C), and around 4 weeks after dissociation neural clusters formed a vast network that connected each cluster by thick neurites on top of the glial-like cells (Figure 3D). These network structures were well maintained at around 8 weeks after dissociation (Figure S4C). IHC showed that SYNAPTOPHYSIN^+^ synaptic connections were formed between TUJ1^+^ components of the neural network (Figure 3E), and astrocyte marker GFAP^+^ cells were also observed (Figure S4D). The expressions of SATB2, CTIP2, FOXG1, and LHX2 were detected in these dissociated neurons, suggesting these cells have the character of cerebral projection neurons (Figures 3F and S4E–S4F′). Regarding neurotransmitters, the existence of VGlut1^+^ glutamatergic neurons, GABA^+^ GABAergic (γ-aminobutyric-acid-releasing) neurons, and TH^+^ dopaminergic neurons were confirmed, although ChAT^+^ cholinergic neurons were not observed (Figures 3G, S4G–S4G″, and S4H). VGlut1^+^ neurons co-expressed the maturation marker CAMK2 (Figures 3H, S4I, and S4I′). Collectively, these data suggested the generation of a self-organizing neuronal network from *in vitro* PSC-derived cerebral organoids that contained mature glutamatergic neurons and GABAergic neurons and showed synapse formation.

Video S5. Phase-Contrast Imaging of the Self-Organized Formation of a Neural Network, Related to Figure 3 The bottom right of the video corresponds to Figures 3A and 3A′, and explanatory text in the Results section.

Additionally, we have succeeded in culturing these *in vitro* derived human neural networks for well over 1 year (Figures S4J–S4M). The expression of SYNAPTOPHYSIN and VGlut1 was evident compared with early stages (Figures S4N–S4O′). TUJ1^+^ neurites were also confirmed at 322 days after dissociation (Figure S4O″), and some of the neurons expressed FOXG1 and CTIP2 (Figure S4P), indicating that an hPSC-derived cerebral network could be maintained *in vitro* for longer than 1 year.

### Individual and Synchronized Activity of Cerebral Organoid-Derived Neural Networks

**Figure 4:**
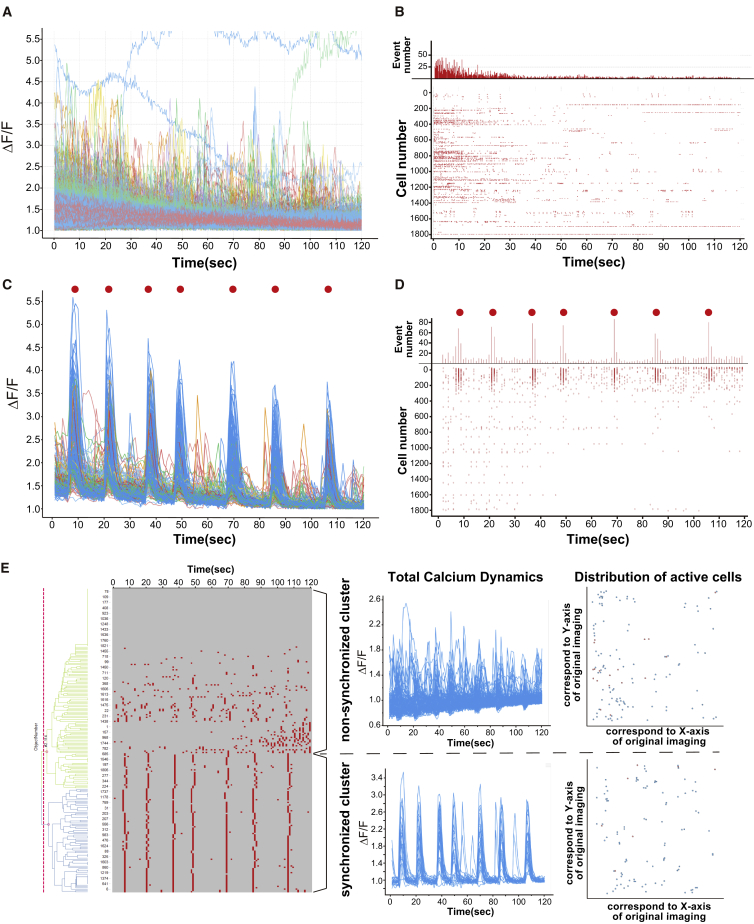
Synchronized Calcium Transients of Cerebral Organoid-Derived Neural Networks (A) The firing pattern of all cells in one field shown by a trace image of the calcium response at 16 days after dissociation. Synchronized bursts were not observed. (B) Raster plot analysis of total cells in one field. Spontaneous calcium transients showed temporally variable activity. (C) The firing pattern of total cells in one field shown by trace images of the calcium response at 30 days after dissociation. Red spots show synchronized bursts. (D) Raster plot analysis of total cells in one field 30 days after dissociation. Spontaneous calcium transients show temporally varied activities and synchronized patterns (red spots). The original images are shown in Video S6. (E) Representative image of the calcium transients and related analyses. Clear separation of synchronized and non-synchronized activity was observed. See also Figure S5 and Video S6.

Next, to evaluate the functionality of the neural networks, we examined intracellular calcium dynamics by confocal live imaging. At early stages after dissociation (16 days), almost all calcium transients showed individual non-synchronized patterns, and only some small groups of cells showed synchronized activity (Figures 4A and 4B; Video S6). Interestingly, at 30 days after dissociation, calcium transients showed a synchronized pattern of network activity but also several individual activities (Figures 4C and 4D; Video S6). Calcium transient data clearly showed synchronized bursts with individual activities (Figure 4C, red spots), and raster plot analysis confirmed the synchronized activity (Figure 4D, red spots). The obtained calcium transient data suggested maturation of the *in vitro* human network activity in a time-dependent manner, consistent with rodent studies (Chiappalone et al., 2006). To further analyze the observed *in vitro* neural network activities, we detected the total calcium transients of all cells in one field (Figures S5A and S5A′) (1,820 cells in total). We then visualized cell activity patterns, made raster plots of the activities, performed cluster analysis of the raster plots, and measured cell distributions (Figure S5B). These analyses successfully visualized synchronized clusters and non-synchronized clusters given the activity patterns and cell distributions from the raster plots (Figure 4E) and could divide the synchronized clusters into several small clusters with information on the cell distribution (Figure S5C).

Video S6. Calcium Imaging of a Neuronal Network Derived from Cerebral Organoids, Related to Figure 4 See also explanatory text in the Results section.

**Figure 5:**
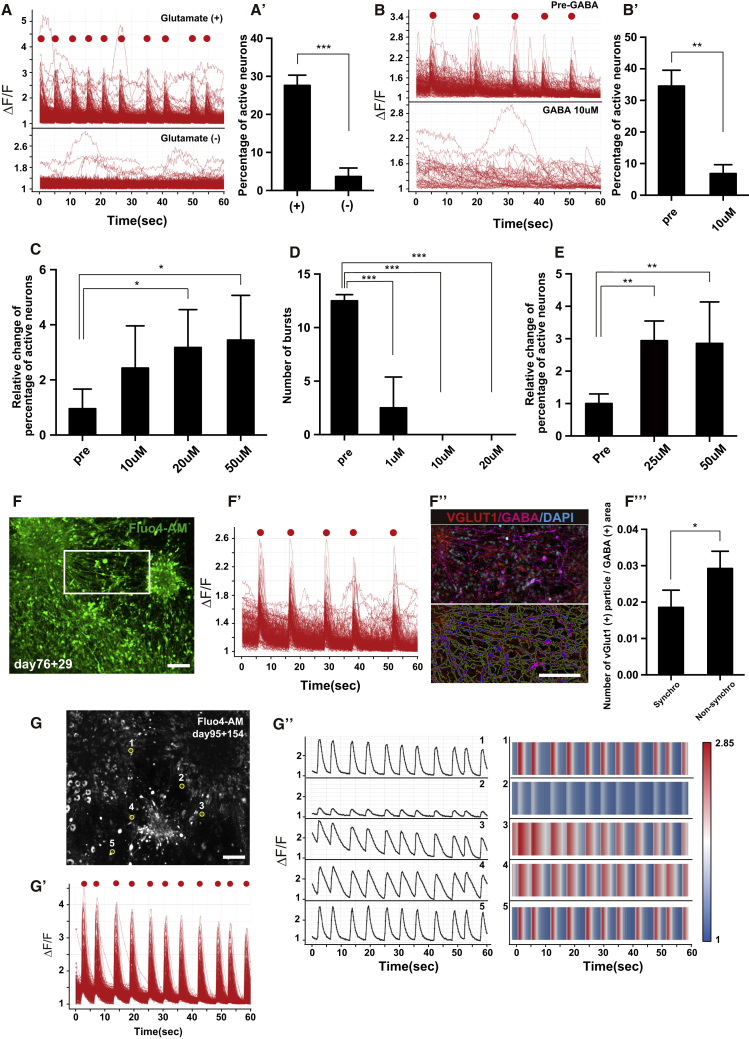
Analysis of Calcium Activity Including Synchronized Bursts (A) Image of calcium transients of all detected cells under glutamate^+^ and glutamate^−^ conditions. (A′) Percentage of active neurons under both conditions. Day 76 + 29 (days for organoid culture + dissociation culture; the same applies hereafter), n = 3. A data point for percentage of active neurons in one field is counted as n = 1. The same applies hereafter in this figure. ^∗∗∗^p < 0.001, unpaired t test. (B) Image of calcium transients of all detected cells before (top) and 1 min after (bottom) GABA treatment (10 μM). (B′) Percentage of active neurons before and after GABA treatment. day 76 + 29, n = 3. ^∗∗^p < 0.01, unpaired t test. (C) Relative change of percentage of active neurons after bicuculline methochloride treatment (10, 20, 50 μM). Day 76 + 30, n = 5. ^∗^p < 0.05, Kruskal-Wallis test. (D) The number of bursts in 60 s of imaging before CNQX treatment and 1 min after CNQX treatment (1, 10, 20 μM). Day 76 + 29, n = 4. ^∗∗∗^p < 0.001, Kruskal-Wallis test. (E) Relative change in percentage of active neurons after D-APV treatment (25, 50 μM). Day 76 + 30, n = 3. ^∗∗^p < 0.01, Kruskal-Wallis test. (F–F‴) Ratio of glutamatergic and GABAergic neurons in synchronized and non-synchronized fields at day 76 + 29. (F) Representative image of a synchronized activity field. (F′) Image of calcium transients in all detected cells in (F). (F‴) IHC of VGlut1 and GABA in the white box in (F) (upper side), and analysis image (lower side). The area surrounded by green indicates the GABAergic neuronal area. The area surrounded by blue indicates VGlut1^+^ particles neighboring the GABA^+^ area. (F‴) The number of VGlut1^+^ particles neighboring the GABA^+^ area is significantly lower in synchronized fields than in non-synchronized fields. The y axis is number of VGlut1^+^ particles/GABA^+^ area (μm^2^). n = 4. ^∗^p < 0.05, unpaired t test. (G–G″) The dataset of calcium imaging at day 95 + 154. (G) Representative image of active neurons. (G′) Image of calcium transients in all detected cells in (G). (G″) The firing pattern of selected cells in (G) shown by the trace image of the calcium response (left side) and a heatmap (right side). Scale bars, 100 μm (F, F″, and G). Error bars in graphs denote SEM.

Next, we examined several aspects of the neural activity including synchronized bursts. The neural activities were observed in the presence of glutamate with synchronized bursts, whereas they were significantly reduced in the absence of glutamate in the same fields (Figures 5A and 5A′). Neural activity was reduced and synchronized bursts were completely blocked by the addition of 10 μM GABA (Figures 5B and 5B′). On the contrary, the activity was significantly upregulated by the addition of 20 or 50 μM (−)-bicuculline methochloride, a GABA_A_ receptor antagonist (Figure 5C). Interestingly, the administration of 6-cyano-7-nitroquinoxaline-2,3-dione (CNQX), a non-NMDA (N-methyl-D-aspartic acid) receptor inhibitor known to block electrical synaptic transmissions, blocked the synchronized bursts at concentrations as low as 1 μM (Figure 5D). The addition of D-(−)-2-amino-5-phosphonopentanoic acid (D-APV), an NMDA receptor inhibitor, upregulated the neural activity (Figure 5E). These analyses suggested that glutamate is the major excitatory transmitter and GABA is the major inhibitory transmitter of the network, and that the activation of non-NMDA receptors generates the synchronized bursts.

We next assessed the difference between the synchronized and non-synchronized fields. Because cooperative interactions between glutamatergic and GABAergic neurons are involved in the initiation of synchronized activity in the neocortex (Murphy et al., 1992), we hypothesized that the proper density of glutamatergic synapses on GABAergic neurons is important for synchronized bursts. To assess this hypothesis, we first performed calcium imaging and obtained four synchronized and four non-synchronized fields (Figures 5F and 5F′). We examined by IHC of VGlut1 and GABA in all fields and assessed the number of VGlut1^+^ particles contacting GABA^+^ neurons (Figure 5F″). This analysis revealed that the number of VGlut1^+^ particles/GABA^+^ area was significantly smaller in the synchronized fields compared with the non-synchronized fields (Figure 5F‴), suggesting that appropriate GABAergic mediation in glutamatergic neurons might be important for synchronized bursts. We detected synchronized bursts up to culture day 249 (Figures 5G–5G″).

### Detection of Drug-Induced Dynamic Changes in Network Activity

**Figure 6:**
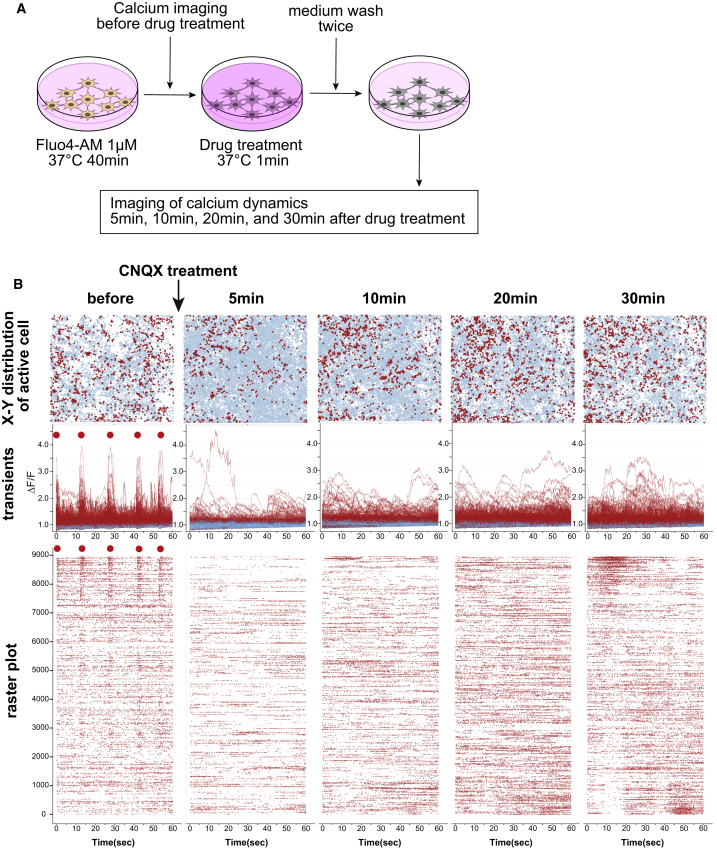
Dynamic Changes in Network Activity during the Drug Load Test with CNQX (A) Schematic of the drug load test (performed on culture day 119, which is 31 days after dissociation). (B) Analysis of the cell distribution, calcium dynamics, and raster plots during the drug load test. The x-y distribution of active cells in the same field is shown in the top row; calcium transients of all active cells are shown in the middle row; and raster plots are shown in the bottom row. Synchronized bursts are shown by red spots. See also Video S7.

Lastly, we sought to examine dynamic changes of the network activity. For this purpose, we tried to assess drug effects by the administration of CNQX, which strongly blocked the synchronized activity. We performed calcium imaging before the drug treatment, then treated the cells with 10 μM CNQX and incubated them at 37°C for 1 min. After washing twice with medium, we obtained 1 min of calcium imaging data at 5, 10, 20, and 30 min after drug treatment in the same field (Figure 6A). Before drug treatment, spontaneous calcium activity with some synchronized activity was detected (Video S7). Sequential calcium imaging showed an almost complete termination of neural activity and remnant slow calcium dynamics in some neurons at 5 min after drug treatment. The calcium transients recovered 10–20 min after drug treatment, and active calcium transients without synchronized activity appeared at 30 min (Video S7). During the drug load test, the difference in active cell distribution between pretreatment and 30 min after drug load could be described (Figure 6B, top panel). Regarding activity patterns, calcium transients and raster plots described synchronized activity with individual activity before treatment, a decrease of activity after drug treatment, and a recovery of activity without synchronization thereafter (Figure 6B, middle and bottom panels).

Video S7. Calcium Imaging during CNQX Load Test, Related to Figure 6 See also explanatory text in the Results section

Collectively, these findings demonstrated a drug-induced dynamic change of the self-organizing activity of cerebral organoid-derived human neural networks.

## Discussion

Here we demonstrated the efficient *in vitro* generation of human cerebral organoids with extended neuroepithelia, performed 3D imaging of the induced cerebral organoids, and assessed the formation of self-organizing neuronal networks including observation of individual and synchronized calcium activities. Regarding functional analysis, our method has strong advantages compared with previously reported organoid studies.

Spontaneous activities of intracellular Ca^2+^ concentrations in synchronized patterns are found in many types of neural tissues *in vivo* and *in vitro*, including the neocortex, hippocampus, neural retina, and spinal cord (Ben-Ari, 2001, Murphy et al., 1992, Spitzer, 1994, Yuste et al., 1992). These spontaneous activities are thought to play a pivotal role in several aspects in neural growth and differentiation such as neuronal migration, axon extension, maturation of glutamatergic synapses, and formation of connection patterning (Chiappalone et al., 2006, Garaschuk et al., 2000, Gomez and Spitzer, 1999, Opitz et al., 2002). In the present study, we found that the synchronized activities are extremely sensitive to low CNQX concentration (1 μM), similarly to the oscillatory calcium waves that start in the posterior cortex and propagate toward the anterior end in immature rat cortex, suggesting the importance of long-range neuronal connections (Garaschuk et al., 2000). Interestingly, we detected synchronized burst activity only in dissociated neuronal networks and not in 3D cultured organoids, even though the culture periods were almost the same in both. One of the ostensible differences between these two models is that the dissociated neuronal network includes several long-range neural fibers. Thus, our findings suggest that synchronized activity regulates the long-range wiring in neural networks. The establishment of mature 3D cerebral organoids as functional models of the *in vivo* cortex could benefit from including these long-distance connections.

Synchronized activity between several neural cell groups are generally thought to encode neuronal information (Silva et al., 2009). Although assessing such neural activity in human model systems has been difficult, mainly due to the lack of functional connectivity (Quadrato and Arlotta, 2017), our approach makes it possible to recognize synchronized activities. In most developing neural networks, GABA is thought to be an important factor for the generation of spontaneous synchronous neuronal activity (Opitz et al., 2002, Voigt et al., 1997), and the development of synchronous calcium activities in neocortical culture depends on the presence of a specific type of GABAergic neuron (Voigt et al., 2001). Consistent with those reports, our data suggested that appropriate GABAergic mediation on glutamatergic neurons may be important for synchronized bursts. In rodent neural tissues, many factors besides GABA, such as gap junctions, glycine, G-protein-coupled receptors, and astrocytes, are thought to drive or regulate the synchronization (Liu et al., 2003, Opitz et al., 2002, Verderio et al., 1999, Yuste et al., 1995). Future examination of these candidates may be needed to study the mechanisms of the neuronal activities that encode and process neural information in human neural networks.

*In vitro* active neuronal networks derived from human cerebral organoids may open new avenues toward drug screenings for complex psychiatric disorders that are characterized by neural dysfunction, as well as screening for adverse effects of putative drugs such as drug-induced epilepsy, and ultimately contribute to the establishment of psychiatric disease models using patient-derived iPSCs (Vadodaria et al., 2018). Thus, they herald a novel paradigm of neuroscientific and neuropharmaceutical research on human brain function and neuropsychiatric disorders.

## Experimental Procedures

### Differentiation Culture of hPSCs

For serum-free floating culture of embryoid body-like aggregates with quick reaggregation (SFEBq) culture, hESCs were dissociated to single cells in TrypLE Express (Invitrogen) containing 12.5 U/mL DNase I (TaKaRa) and 10 μM Y-27632, and quickly reaggregated using low-cell-adhesion-coated 96-well plates with V-bottomed conical wells (Sumilon PrimeSurface plate; Sumitomo Bakelite) in differentiation medium (9,000 cells per well, 100 μL) containing 50 μM Y-27632 under 5% CO_2_. The addition of Y-27632 was stopped after day 3. The differentiation medium was Glasgow’s minimal essential medium supplemented with 0.1 mM non-essential amino acids, 1 mM pyruvate, 0.1 mM 2-mercaptoethanol, 20% (v/v) knockout serum replacement, 100 U/mL penicillin, and 100 μg/mL streptomycin. Defining the day on which the SFEBq culture was started as day 0, 5 μM SB431542 (transforming growth factor β inhibitor; Tocris) and 3 μM IWR1e (Wnt inhibitor; Calbiochem) were added to the culture from day 0 to day 18. Half the medium was changed once every 3 days.

At day 18, the floating aggregates were transferred from a 96-well plate to a 100-mm Ezsphere dish (Iwaki, cat. #4020-900) and further cultured in suspension using DMEM/F12, GlutaMAX medium (Thermo Fisher) supplemented with N2 supplement (Thermo Fisher), Chemically Defined Lipid Concentrate (Invitrogen), 0.25 mg/mL fungizone (Gibco), 100 U/mL penicillin, and 100 μg/mL streptomycin under 37°C, 40% O_2_/5% CO_2_ conditions.

For long-term culture of cerebral tissues, the aggregates were cultured under the following conditions. At day 35, the aggregates were cut into halves or thirds with fine forceps under a dissecting microscope for the prevention of cell death in the central portions of the aggregates, and were cultured using a Lumox dish (Sarstedt; high O_2_ penetration) after day 50. Medium change was performed once every 3 days.

For differentiation from hiPSCs, the cells were dissociated into single cells by treatment with Accumax (Innovative Cell Technologies) and quickly reaggregated using low-cell-adhesion-coated 96-well plates with V-bottomed conical wells (Sumilon PrimeSurface plate; Sumitomo Bakelite) in differentiation medium (9,000 cells per well, 100 μL) containing 50 μM Y-27632 under 5% CO_2_. The addition of Y-27632 was stopped after day 3, and the following steps were the same as with hESCs.

### Neuronal Dissociation Culture Method

For the dissociation culture, cells were dissociated from aggregates using a Neural Tissue Dissociation Kit (Sumitomo Bakelite, MB-X9901) on days 70–100 and plated onto poly-D-lysine/laminin/fibronectin-coated glass dishes or Cell Desk LF-1 (Sumitomo Bakelite, MS-92132) at a density of 300,000–500,000 cells/cm^2^ in DMEM/F12, GlutaMAX medium (Thermo Fisher) supplemented with N2 supplement (Thermo Fisher), Chemically Defined Lipid Concentrate (Invitrogen), 20 ng/mL brain-derived neurotrophic factor, 20 ng/mL glial cell line-derived neurotrophic factor, 0.25 mg/mL fungizone (Gibco), 100 U/mL penicillin, 100 μg/mL streptomycin, and 1% fetal bovine serum. Thereafter, one-half of the medium volume was changed every 2–3 days.

### Immunohistochemistry

Immunohistochemistry (IHC) was performed as previously reported (Sakaguchi et al., 2015). In brief, samples were fixed in 4% paraformaldehyde at 4°C for 20–30 min, permeabilized with 0.05% Triton X-100 in PBS for 45 min at room temperature, incubated with block reagent (2% skim milk in PBS) at room temperature, and incubated with primary antibodies (4°C overnight) followed by incubation with secondary antibodies conjugated with Alexa 488, 594, 647, and DAPI (at room temperature for 2 h). For the staining of GABA, 0.05% glutaraldehyde (Nacalai Tesque) was included in the fixation stage. The antibodies were used at the following dilutions: LHX2 (goat, 1:200, Santa Cruz, sc-19342), FOXG1 (rabbit, 1:2000, TaKaRa, M227), SOX2 (goat, 1:250, Santa Cruz, sc-17320), CTIP2 (rat, 1:5,000, Abcam, ab18465), TBR1 (rabbit, 1:500, Abcam, ab31940), PAX6 (mouse, 1:500, Becton Dickinson, 561462), SATB2 (mouse, 1:150, Abcam, ab51502), TBR2 (chicken, 1:1,000, Millipore, AB15894), pVIM (mouse, 1:500, Abcam, ab22651), calretinin (rabbit, 1:200, Abcam, ab16694), SYNAPTOPHYSIN (guinea pig, 1:1,000, Zymed, 18-0130), TUJ1 (rabbit, 1:2,000, Covance, PRB-435P), GABA (mouse, 1:250, Millipore, MAB316), VGlut1 (rabbit, 1:1,000, Synaptic systems, 135-303), ChAT (goat, 1:200, Millipore, AB144P), GFAP (mouse, 1:200, Sigma, G3893), CAMKII (mouse, 1:500, Abcam, ab22609), and NCAD (mouse, 1:1,000, BD, 610920). Counter nuclear staining was performed with DAPI (Molecular Probes).

### Calcium Imaging of the Neuronal Network

For calcium dye loading, the cells were incubated with 1 μM Fluo4-AM solution (Invitrogen) for 40 min at 37°C. Excess dye was removed by washing with culture medium. Imaging was carried out at 37°C and 5% CO_2_ using a confocal quantitative image cytometer (CQ1; Yokogawa). Fluo4-AM dyes were excited at 488 nm using a diode-pumped solid-state laser (OBIS488L; Coherent), and fluorescence emission was viewed through a dry objective lens (UPLSAPO10× [NA 0.4], UPLSAPO20X [NA. 0.75]; Olympus). Time-lapse image sequences were acquired at 200-ms intervals for 2 min (Figures 4A and 4B; Video S6), 1-s intervals for 2 min (Figures 4C and 4D; Video S6), and 200-ms intervals for 1 min (Figures 5 and 6; Video S7). Images were processed using CQ1 Measurement (Yokogawa) for the sequential detection of each single-cell fluorescence during total imaging and the cell distribution, and processed using custom-designed Spotfire (Data Visualization & Analytics Software—TIBCO Spotfire, http://spotfire.tibco.com/) programs for the simultaneous visualization of cell activity patterns, creation of raster plots of the activities, performance of cluster analysis, and arrangement of the cell distribution. The fluorescence change over time is defined as ΔF/F = (F − F_basal_)/F_basal_, where F is the fluorescence at any time point and F_basal_ is the minimum fluorescence of each cell. A neuron was considered active if calcium transients were observed at least once in total imaging. For pharmacological experiments, GABA (10 μM), bicuculline methochloride (10–50 μM), CNQX (1–20 μM), or D-APV (25–50 μM) was applied by bath application, and the cells were incubated at 37°C for 1 min. In the experiments shown in Figure 6, after two washes with medium, 1-min calcium imaging data were taken at 5, 10, 20, and 30 min after drug treatment in the same field.

### Calcium Imaging of Cerebral Organoids

For calcium dye loading, the cells were incubated with 1 μM Fluo4-AM solution for 3 h at 37°C. Excess dye was removed by washing with culture medium. Imaging was carried out at 37°C and 5% CO_2_ using a two-photon microscope (A1R MP^+^; Nikon). Fluo4-AM dyes were excited at 820 nm or 830 nm using Mai Tai DeepSee (Spectra Physics), and fluorescence emission was viewed through an X20 dry objective lens (MRD70200; Nikon, NA 0.75). Time-lapse image sequences were acquired at 1-s intervals for 2 min. Images were processed using ImageJ software and custom-designed Spotfire programs.

### Statistical Analysis

Statistical tests were performed with PRISM software (GraphPad, version 5). Statistical significance was tested with unpaired t test (non-parametric) for two-group comparisons, one-way ANOVA test (parametric versus control group) for multiple-group comparisons, and Kruskal-Wallis test (non-parametric versus control group) for multiple-group comparisons.

### Code Availability

This study used custom-designed software which will be available to interested investigators upon reasonable request.

## Author Contributions

H.S. and J.T. designed the project; H.S., Y.O., T.A., and T.M. performed the research; H.S., Y.O., T.A., T.M., and N.O. analyzed the data; H.S. and J.T. obtained grants for the research; T.M. and O.N. contributed the analytical tools for calcium imaging by discussing details with H.S.; H.S. and S.K. performed the imaging of cerebral organoids by two-photon microscopy; H.S. wrote the paper with feedback from all authors.

## Accession Numbers

The datasets generated during this study are available from the corresponding author (H.S.) upon reasonable request.

## Supplemental Information

Document S1. Supplemental Experimental Procedures and Figures S1–S5

Document S2. Article plus Supplemental Information
