# Supplementary material for: Self-Organized Synchronous Calcium Transients in a Cultured Human Neural Network Derived from Cerebral Organoids
Source: Stem Cell Reports. 2019 Jun 27;13(3):458–73. doi: 10.1016/j.stemcr.2019.05.029 (PMC6739638; doi:10.1016/j.stemcr.2019.05.029)
Supplement: Document S1. Supplemental Experimental Procedures and Figures S1–S5 [file mmc1.pdf]

Stem Cell Reports, Volume *13*

Supplemental Information

Self-Organized Synchronous Calcium Transients in a Cultured Human Neural Network Derived from Cerebral Organoids

Hideya Sakaguchi, Yuki Ozaki, Tomoka Ashida, Takayoshi Matsubara, Naotaka Oishi, Shunsuke Kihara, and Jun Takahashi

Supplemental Experimental Procedures

Maintenance culture of human pluripotent stem cells (hPSCs)

This study was approved by the ethics committees of Kyoto University, Kyoto, Japan. Human embryonic stem cells (hESCs) (KhES-1) were used according to the hESC research guidelines of the Japanese government. hESCs were maintained and cultured as previously described (Kadoshima et al., 2013, Sakaguchi et al., 2015). In brief, hESCs were maintained on a feeder layer of mouse embryonic fibroblasts (MEFs) inactivated by 10 μg/ml mitomycin C treatment in DMEM/F12 (Sigma) supplemented with 2 mM glutamine, 0.1 mM nonessential amino acids (Invitrogen), 5 ng/mL recombinant human basic FGF (Wako), 0.1 mM 2-mercaptoethanol (2-ME), 20% (vol/vol) Knockout Serum Replacement (KSR; Invitrogen), 50 U/mL penicillin, and 50 μg/mL streptomycin under 2% CO_2. For passaging, hESC colonies were detached and recovered en bloc from the feeder layer by treating them with 0.25% (wt/vol) trypsin and 1 mg/mL collagenase IV in PBS containing 20% (vol/vol) KSR and 1 mM $CaCl_2$ at 37 °C for 8 min. The detached hESC clumps were broken into smaller pieces by gentle pipetting. The passages were performed at a 1:4–1:8 split ratio every 4-5 days.

hiPSCs (1231A3) were maintained and cultured as previously described (Nakagawa et al., 2014). Briefly, hiPSCs were maintained on LN511-E8-coated dishes with StemFit medium (Ajinomoto). For passaging, the cells were dissociated into single cells by treatment with Accumax (Innovative Cell Technologies) and replated at a density of 1.0-1.5 × 10^4 cells into each well of a 6-well plate. 10 μM Y-27632 was added for the first day. The medium was changed every day. The passages were performed every 7 days.

Immunohistochemistry quantification

All cell counts and measurements were performed using ImageJ, Adobe Photoshop and Microsoft PowerPoint. In Figure 2h and Extended Figure 3d, the thickness of the total epithelium (defined as the length between the most inner side of the Pax6$^+$ region and the surface of the epithelium), deep layer (defined as dense Ctip2$^+$ or Tbr1$^+$ regions), and upper layer (defined as dense Satb2$^+$ regions) were measured. In Figure S3e, the distance between the apical side and p-vimentin$^+$/Sox2$^+$ cells was defined as the length between p-vimentin$^+$/Sox2$^+$ cells and the most inner side of the Sox2$^+$ VZ layer.

Tissue elasticity measurement

The tissue elasticity of hESC-derived cell aggregates was examined by SPM-9700HT (Shimadzu) with Nano 3D mapping mode (one type of contact mode) using an AFM cantilever. The used cantilever was AC200; spring constant: 9N/m (Olympus). The cell aggregates were pressed by the force probe, and

force-curve data were taken. The analysis was done with the JKR method of Nano 3D mapping mode in SPM-9700HT. The measurement was done in PBS on the surface of cell aggregates (culture day 1) attached on a petri dish by minimum adhesive agent using a stereoscopic microscope in both Y20 and Y50 conditions (n=9 from 3 aggregates for each condition). All measurements were performed at room temperature.

Tissue rarefaction by CLARITY system

For tissue rarefaction, we adopted the commercial-based CLARITY system (LifeCamvas Technologies & Bio Research Center) and modified the original method to optimize for organoid rarefaction. In this system, organoids were first fixed with 4% PFA at 4°C for 1 hour, then incubated in hydrogel monomer solution consisting of 4% acrylamide (161-0140; Bio-Rad), 0.25% azoinitiator (VA-044; Wako Chemicals), and 1X PBS (70011-044; Invitrogen) in UltraPure water (all wt/vol) at 4°C for O/N, followed by gentle shaking in a 37°C water bath for 2 hours under negative pressure using a vacuum pump. The tissues were recovered from the polymerized solution, and pieces of gel or solution were carefully removed from the tissue using paper towel. The tissues were washed with Smart Clear Clearing Buffer type A (LifeCamvas Technologies, Cat#: SC-B2001) at 37°C for O/N with gentle shaking. Then, stochastic electrotransport was performed using a SmartClear II device (LifeCamvas Technologies) at 1000 mA, 50 V, and 37°C for 2 hours. Lastly, tissues were incubated in EasyIndex Optical Clearing Solution (LifeCamvas Technologies, Cat#: EI-Z1011) at 37°C with gentle shaking, and after several hours, the tissues became totally transparent.

3D immunohistochemistry and imaging

IHC of whole aggregates was performed as follows. After the tissue rarefaction step, the samples were permeabilized with 1% triton/PBS for 1 hour at room temperature. After PBS wash 3 times, aggregates were incubated with block reagent (2% skim milk in PBS) 3 hours at RT. The primary antibody was applied and incubated for two days at 4°C and washed with 0.05% tween20 (2 hours, 3 times each). The second antibody was applied and incubated for O/N at 4°C. Lastly, aggregates were incubated in EasyIndex Optical Clearing Solution (LifeCamvas Technologies, Cat#: EI-Z1011). Stained samples were analyzed with a light sheet microscope (Zeiss) or confocal microscope (YOKOGAWA). The 3D imaging in Figure 2c and 2d was made by ArivisVision software and ImageJ software, respectively.

Bright field view time-lapse imaging of cell aggregation

Differentiation culture of hPSCs was performed as described above with V-bottomed conical wells (Sumilon PrimeSurface plate; Sumitomo Bakelite) in differentiation medium (9,000 cells per well, 100 μL)

containing 20 μM or 50 μM Y-27632 under 5% CO_2. The original imaging data was taken by IncuCyte S3 Spheroid Software Module (Essen Bioscience) every 30 min for 3 days during tissue differentiation under both Y20 and Y50 conditions. Images were processed by WCIF ImageJ software.

Phase contrast time-lapse imaging and neurite extension analysis

Dissociated cerebral organoid-derived cells were seeded at 50×10^4 cells in each well of a 24-well plate (TPP, Cat#: 92424) coated by poly-D-lysine/laminin/fibronectin, and cultured at 37 °C, 5% CO_2 in the same media used for the dissociation culture. Phase contrast microscopic images were automatically taken using the IncuCyte S3 Live Cell Imaging System every 15 min for 6 days. The condition of automatic detection of neurite length using IncuCyte's NeuroTrack software was optimized by analyzing sample pictures at days 1, 2, and 5. After the appropriate detection of neurites and cell bodies was confirmed, automatic analysis of neurite length was performed. Images were processed by WCIF ImageJ software.

Supplemental Reference

Nakagawa, M., Taniguchi, Y., Senda, S., Takizawa, N., Ichisaka, T., Asano, K., Morizane, A., Doi, D., Takahashi, J., Nishizawa, M. et al. (2014). A novel efficient feeder-free culture system for the derivation of human induced pluripotent stem cells. Scientific Reports 4, 3594.

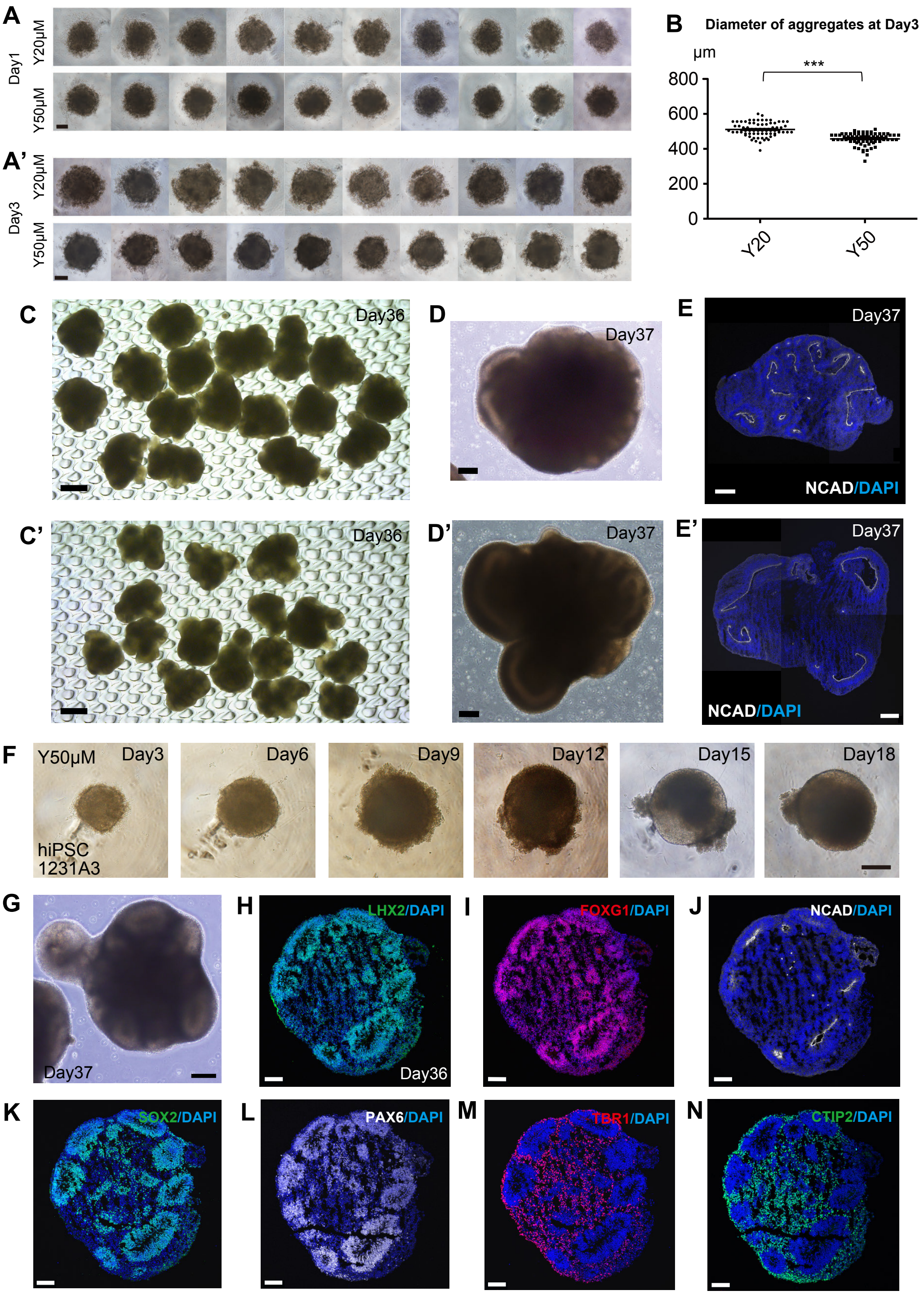

Figure S1 | Generation of cerebral organoids with elongated epithelium from hPSCs. Related to Figure 1

(**A-A'**) Ten representative phase contrast images of cell aggregates under Y20 condition (Y-27632 20 μM)

or Y50 condition (Y-27632 50 μM) on day 1 (**A**, from 6 independent examinations) and day 3 (**A'**, from 7 independent examinations). (**B**) Diameter of aggregates on day 3. The diameter is significantly smaller under Y50 condition. Sample number of each condition is 75 aggregates from 7 independent experiments. ***$p<0.001$, unpaired t-test. (**C-C'**) Low magnification bright field view images of Y20 (**C**) and Y50 conditions (**C'**) on day 36. (**D-D'**) High magnification phase contrast images of Y20 (**D**) and Y50 conditions (**D'**) on day 37. (**E-E'**) IHC for NCAD of day 37 aggregates under each condition. A more elongated epithelium was formed under Y50 condition. (**F-G**) Phase contrast images of hiPSC (1231A3)-derived aggregation under Y50 condition on days 3, 6, 9, 12, 15, 18, and (**G**) 37. (**H-N**) IHC of cortical related markers FOXG1, LHX2, NCAD, SOX2, PAX6, TBR1, and CTIP2 for hiPSC-derived aggregates under Y50 condition on day 36. Scale bars, 1000 μm in (**C-C'**), 200 μm in (**A-A', D-G**) and 100 μm in (**H-N**). Bars in graph, SEM. Nuclear counter-staining (blue), DAPI.

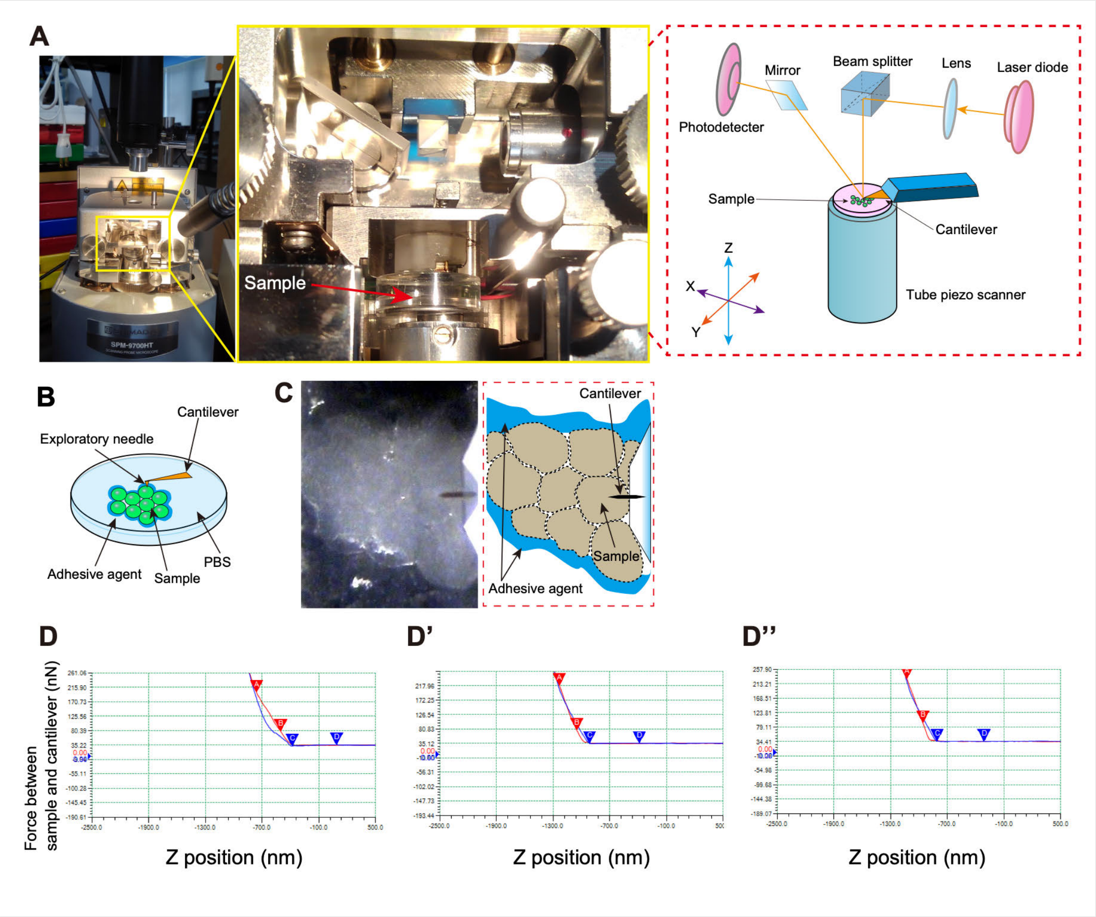

Figure S2 | Elasticity examination of cell aggregation using atomic force microscopy. Related to Figure 1

(**A**) Picture and schematic of the atomic force microscope (AFM) system. (**B-C**) Picture and schematic of a sample in PBS under elasticity examination. (**D-D''**) Three representative images of force curve reflecting elasticity.

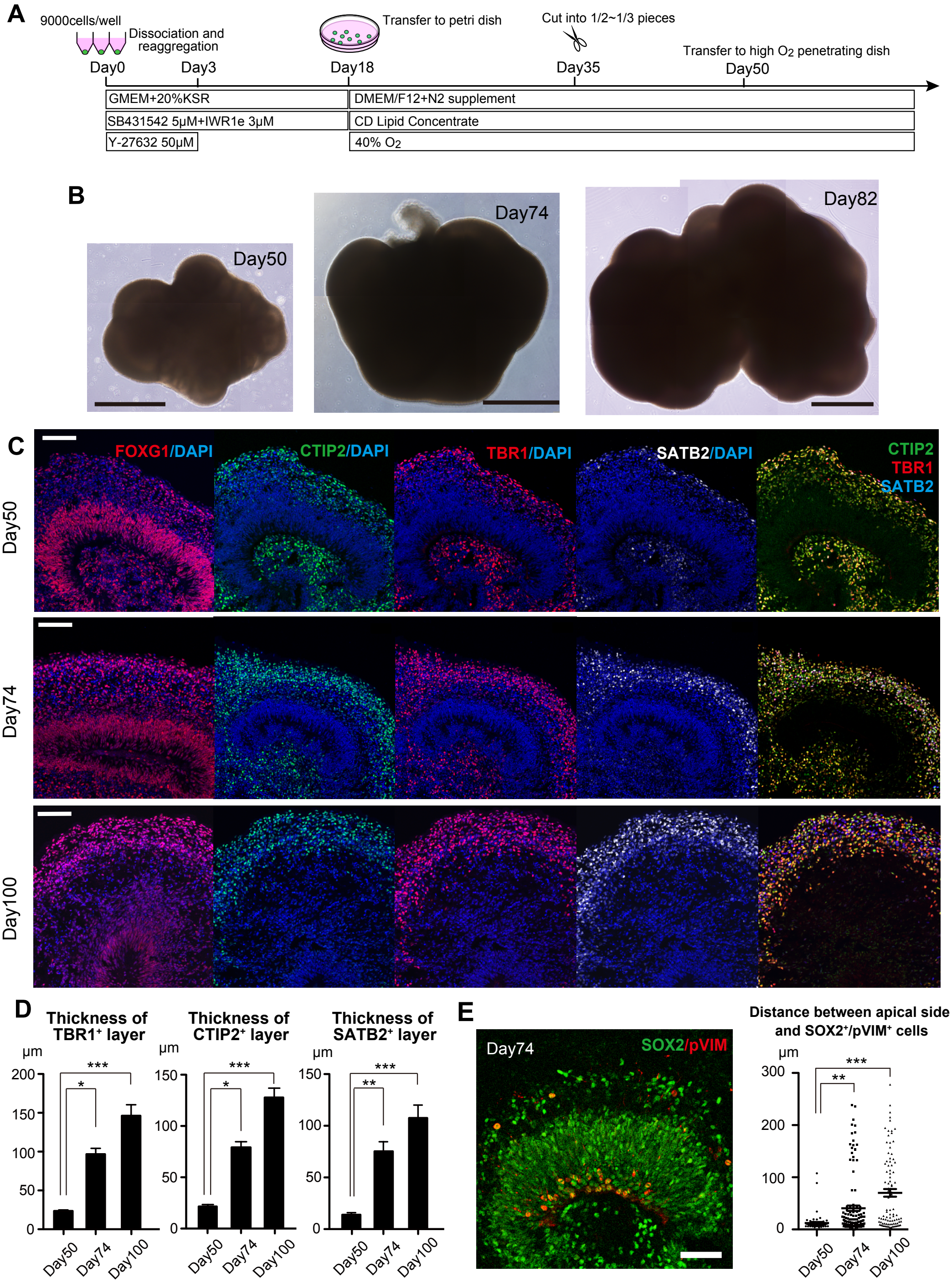

Figure S3 | Generation of cerebral organoids and recapitulation of developmental process by long-term culture. Related to Figure 2

(**A**) Schematic for long-term culture of cerebral organoids. (**B**) Phase contrast images of cerebral organoids

induced under Y50 condition on days 50, 74 and 82. (**C**) IHC for FOXG1, CTIP2, TBR1, and SATB2 of day 50, 74, and 100 aggregates. The layer expressing CTIP2, TBR1, and SATB2 become apparent and thicker with progression of the culture period. (**D**) Thickness of the $TBR1^+$, $CTIP2^+$ and $SATB2^+$ cell layer in day 50, 74, and 100 epithelia. The thickness significantly increased on days 74 and 100 compared with day 50 in every quantification. n=10 from 6 samples for day 50, and n=10 from 5 samples for days 74 and 100. One cricoid-like epithelium is counted as n=1. *p<0.05, **p<0.01, ***p<0.001, one way ANOVA. (**E**) Representative image of IHC for pVIM and SOX2 at day 74, and distance between the apical side and $SOX2^+$/$pVIM^+$ cells on days 50, 74, and 100. The distance was significantly longer on days 74 and 100 compared with day 50. n=66 from 4 samples for day 50, n=127 from 5 samples for day 74, and n=94 from 5 samples for day 100. **p<0.01, ***p<0.001, one way ANOVA. Scale bars, 1000 μm in (**B**), 100 μm in (**C**), and 50 μm in (**E**). Bars in graph, SEM. Nuclear counter-staining (blue), DAPI.

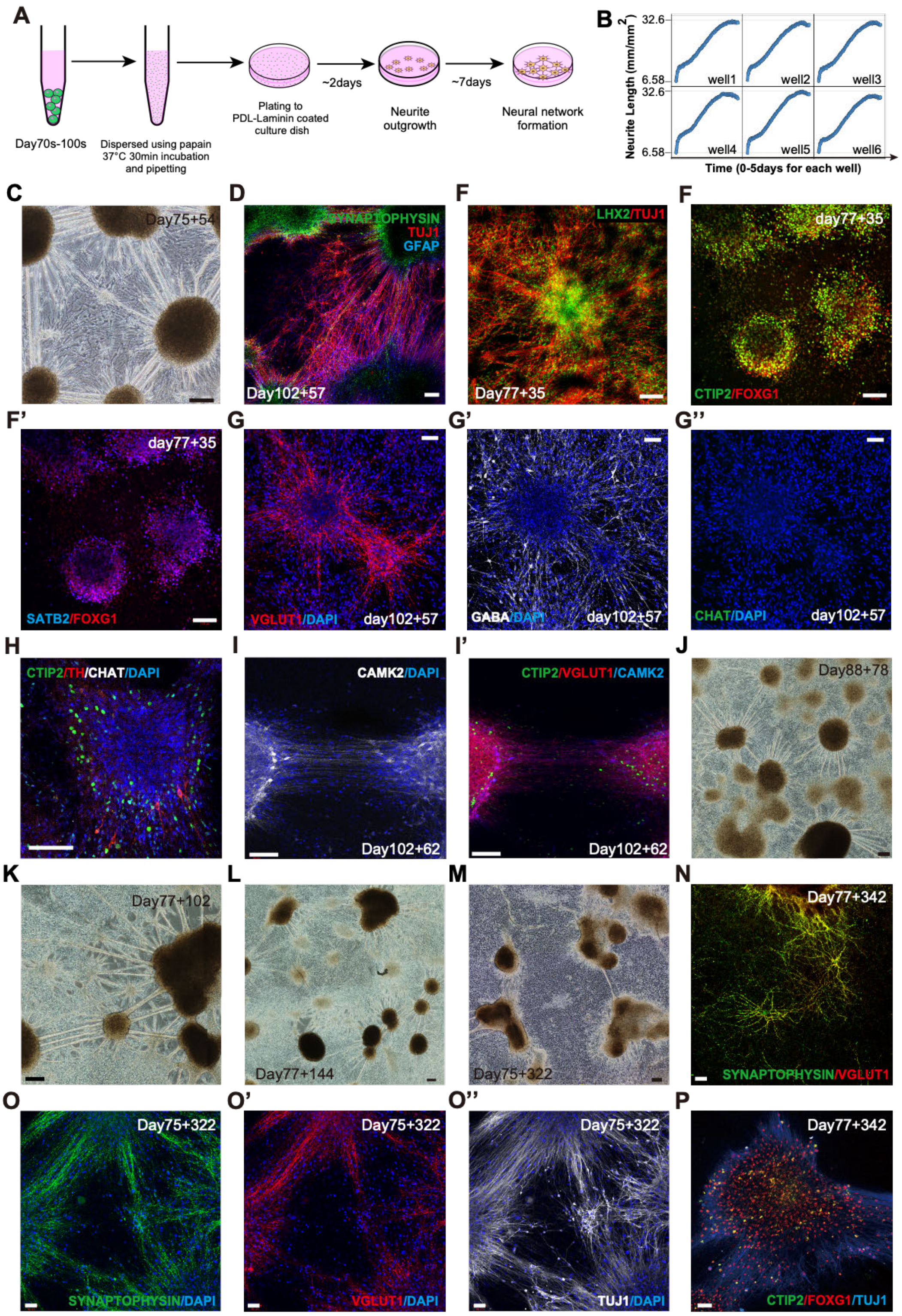

Figure S4 | Self-organized neural network formation by dissociation of cerebral organoids and its long-term culture for over 1 year. Related to Figure 3

(**A**) Schematic of the dissociation culture of cerebral organoids. (**B**) Neurite extension analysis at 15-minute

intervals for 5 days. The data are the average values of 16 fields from 1 well of a 24 well dish taken by a tenfold objective lens. The data of well 6 correspond to Figure 3b. All 6 wells showed robust neurite extension. (**C**) Phase contrast image of an organoid 54 days after dissociation. (**D**) IHC for TUJ1, SYNAPTOPHYSIN, and GFAP 57 days after dissociation. (**E**) IHC for LHX2 and TUJ1 35 days after dissociation. (**F-F'**) IHC for CTIP2 and FOXG1 (**F**), and SATB2 and FOXG1 (**F'**) 35 days after dissociation. (**G-G''**) IHC of the same field for VGLUT1 (**G**), GABA (**G'**), and CHAT (**G''**) 57 days after dissociation. (**H**) IHC for CTIP2, TH, and CHAT 40 days after dissociation. (**I-I'**) IHC of the same field for CAMK2, VGLUT1, and CTIP2 62 days after dissociation. (**J-M**) Phase contrast images of a long-term culture of a dissociated organoid 78 days (**J**), 102 days (**K**), 144 days (**L**), and 322 days (**M**) after dissociation. (**N**) IHC for SYNAPTOPHYSIN and VGLUT1 342 days after dissociation. (**O-O''**) IHC of the same field for SYNAPTOPHYSIN (**O**), VGLUT1 (**O'**), and TUJ1 (**O''**) 322 days after dissociation. (**P**) IHC for CTIP2, FOXG1, and TUJ1 342 days after dissociation. Scale bars, 200 μm in (**C, J-M**), 100 μm in (**D-F', H-I'**), and 50 μm in (**G-G'', N-P**). Nuclear counter-staining (blue), DAPI.

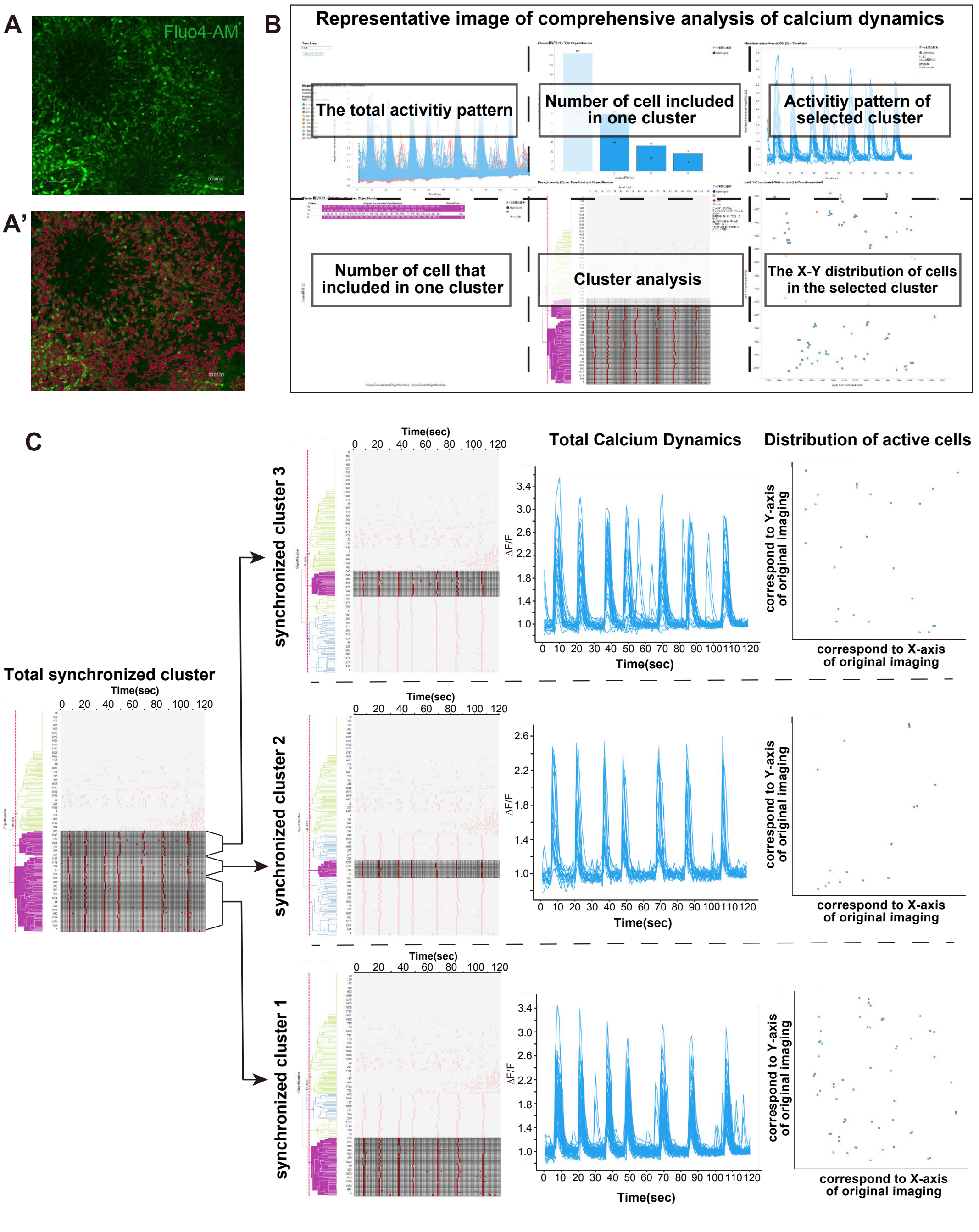

Figure S5 | Representative of calcium imaging analysis. Related to Figure 4

(**A-A'**) Representative image of calcium imaging (**A**), and the ROI setting (**A'**) (day 118, 4 weeks after dissociation). (**B**) Representative image of comprehensive analysis of calcium dynamics. The total activity

pattern, cluster analysis, number of cells included in one cluster, activity pattern of the selected cluster, and the X-Y distribution of cells in the selected cluster are shown in one screen. (**C**) Representative image of the analysis of calcium transients of the selected cluster. The synchronized activity was divided into 3 clusters, with the raster plot, calcium dynamics, and cell distribution determined for each.

Supplemental Movie Legends

Supplemental Movie 1 | Bright field view imaging of cell aggregation process from days 0 to 3. Related to Figure 1

The original imaging data were taken by using the IncuCyte S3 Live Cell Imaging System every 30 min for 3 days during tissue differentiation under Y20 (left) or Y50 (right) conditions. Images were processed by WCIF ImageJ software. Scale bar, 400 μm.

Supplemental Movie 2 | 3D imaging of a whole organoid at day 74 using light sheet microscopy. Related to Figure 2

Movie corresponds to Figure 2c.

Supplemental Movie 3 | Z-stack imaging of a whole organoid at day 100 using confocal microscopy. Related to Figure 2

Movie corresponds to Figure 2d.

Supplemental Movie 4 | Calcium imaging of a cerebral organoid using multi-photon microscopy at days 76, 90, and 104. Related to Figure 2

Movie corresponds to Figure 2i-k.

Supplemental Movie 5 | Phase contrast imaging of the self-organized formation of a neural network. Related to Figure 3

The bottom right of the movie corresponds to Figure 3a and a'.

Supplemental Movie 6 | Calcium imaging of a neuronal network derived from cerebral organoids. Related to Figure 4

Movie corresponds to Figure 4. Imaging of intracellular calcium dynamics at 2 weeks (16 days) and 4 weeks (30 days) after dissociation are shown.

Supplemental Movie 7 | Calcium imaging during CNQX load test. Related to Figure 6

Movie corresponds to Figure 6.
